# Single-cell profiling identifies LIN28A mRNA targets in the mouse pluripotent-to-2C-like transition and somatic cell reprogramming

**DOI:** 10.1016/j.jbc.2024.107824

**Published:** 2024-09-27

**Authors:** Jieyi Hu, Jianwen Yuan, Quan Shi, Xiangpeng Guo, Longqi Liu, Miguel A. Esteban, Yuan Lv

**Affiliations:** 1 Laboratory of Integrative Biology, Guangzhou Institutes of Biomedicine and Health, Chinese Academy of Sciences, Guangzhou, China; 2 University of Chinese Academy of Sciences, Beijing, China; 3 College of Life Sciences, University of Chinese Academy of Sciences, Beijing, China; 4 BGI Research, Shenzhen, China; 5 3DC STAR Lab, BGI CELL, Shenzhen, China; 6 Laboratory of Genomics and Molecular Biomedicine, Department of Biology, University of Copenhagen, Copenhagen, Denmark; 7 Centre for Genomic Regulation (CRG), Barcelona Institute of Science and Technology (BIST), Barcelona, Spain; 8 Universitat Pompeu Fabra (UPF), Barcelona, Spain; 9 Bioland Laboratory (Guangzhou Regenerative Medicine and Health Guangdong Laboratory), Guangzhou, China; 10 BGI Research, Hangzhou, China; 11 Shanxi Medical University-BGI Collaborative Center for Future Medicine, Shanxi Medical University, Taiyuan, China

**Keywords:** LIN28A, RNA-binding proteins, TRIBE, RNA-protein interactions, reprogramming, pluripotency, totipotency

## Abstract

RNA-binding proteins (RBPs) regulate totipotency, pluripotency maintenance, and induction. The intricacies of how they modulate these processes through their interaction with RNAs remain to be elucidated. Here we employed Targets of RBPs Identified By Editing (TRIBE) with single-cell resolution (scTRIBE) to profile the mRNA targets of the key pluripotency regulator LIN28A in mouse embryonic stem cells (ESCs), 2-cell embryo-like cells (2CLCs), and somatic cell reprogramming. LIN28A is known to act by controlling the maturation of the *let-7* microRNA, but, in addition, it binds to multiple mRNAs and influences their stability and translation efficiency. However, the mRNA targets of LIN28A in 2CLCs and reprogramming are unclear. Through quantitative single-cell analysis of the scTRIBE dataset, we observed a marked increase in the binding of LIN28A to mRNAs of ribosome biogenesis factors and a selected group of totipotency factors in 2CLCs within ESC cultures. Our results suggest that LIN28A extends the half-life of at least some of these mRNAs, providing new insights into its role in the totipotent state. We also uncovered the distinct trajectory-specific LIN28A-mRNA networks in reprogramming, helping explain how LIN28A facilitates the mesenchymal-to-epithelial transition and pluripotency acquisition. Our study not only clarifies the multifunctional role of LIN28A in these processes but also highlights the importance of decoding RNA-protein interactions at the single-cell level.

Mammalian pluripotency is the ability of a cell to develop into any cell type composing the body. It is a transient property of the epiblast of the developing blastocyst and can be sustained in culture in the form of embryonic stem cells (ESCs) or induced by the reprogramming of somatic cells to induced pluripotent stem cells (iPSCs) (1). Interestingly, mouse ESC cultures are inherently metastable, containing a small proportion of cells (0.5–1%) that resemble the totipotent 2-cell (2C) stage in embryogenesis; these cells are termed 2C-like cells (2CLCs) (2). Understanding the molecular mechanisms governing the maintenance and induction of pluripotency and the emergence of totipotent-like cells *in vitro* is of fundamental relevance for developmental biology and regenerative medicine.

RNA-binding proteins (RBPs) constitute approximately 10% to 15% of the total proteome in the cell and have long been recognized as key regulators of cell fate decisions including pluripotency regulation and reprogramming (3, 4). RBPs are broadly involved in multiple aspects of RNA metabolism including splicing, stability, and translation, but also exert their roles through non-classical (RNA metabolism-independent) mechanisms (5). Among the RBPs involved in pluripotency and reprogramming, LIN28A stands out as a pivotal player (6). A major mechanistic role of LIN28A is the inhibition of *let-7* microRNA (miRNA) maturation, but in addition, it regulates the stability and translation efficiency of thousands of mRNAs (7). However, the landscape of LIN28A mRNA targets in ESCs and the multiple stages of reprogramming are poorly understood, hindering a comprehensive understanding of its functions. Interestingly, LIN28A has also been proposed to act as a barrier for generating 2CLCs from pluripotent stem cells (ESCs or iPSCs) (8). On the one hand, the interaction of LIN28A with small nucleolar RNAs (snoRNAs), ribosomal RNAs (rRNAs), nucleolar factors (*e.g.,* NCL), ribosome biogenesis factors (*e.g.*, GNL2 and NOP14) and ribosomal subunits (*e.g.*, RPL5 and RPS6) regulates nucleolar structure and promotes rRNA biogenesis to suppress the totipotent state. On the other hand, the LIN28A-NCL-TRIM28 complex represses transcription of the totipotent transcription factor *Dux* and binds to rDNA loci to promote rRNA transcription. Whether other mechanisms, specifically potential mRNA targets of LIN28A in 2CLCs, are involved is unclear.

Methods to identify RNA targets of RBPs include two main approaches (9). Classical approaches such as crosslinking and immunoprecipitation involve the purification of RBP-RNA complexes for sequencing (CLIP-sequencing, CLIP-seq) (10). This strategy requires a large amount of starting material due to limited cross-linking efficiency and problems with antibody recognition efficiency. Previous studies have used this method to identify the transcriptome-wide RNA targets of LIN28A in mouse ESCs (11). Besides the above-mentioned problems, a caveat of this work is that ESC cultures are heterogeneous, overlooking for example the small population of 2CLCs (12). The same principle applies to somatic cell reprogramming, where only a fraction of the starting cells reaches pluripotency through a multi-step process with different intermediate cell states (13). The second approach fuses RNA editing enzymes with RBPs and includes for example TRIBE (Targets of RBPs Identified By Editing) and STAMP (Surveying Targets by APOBEC-Mediated Profiling) (14, 15). This strategy edits the RNA of RBP-bound regions, which can be identified through sequencing. By bypassing less efficient steps, this methodology enables the detection of RBP targets in a small number of cells and even at the single-cell level (14, 15, 16).

Here, we used TRIBE coupled to single-cell RNA sequencing (scRNA-seq) (hereafter referred to as scTRIBE) to explore the cell type-specific mRNA targets of LIN28A in the mouse pluripotent-to-totipotent transition within ESCs and somatic cell reprogramming. We discovered that LIN28A interacts with the mRNAs of multiple ribosome biogenesis factors, which is associated with their increased mRNA stability in 2CLCs, and with the mRNA of 2C-stage-embryo enriched factors such as the *Zscan4* family of transcription factors (*Zscan4a*, *Zscan4d*). Additionally, we identified distinct LIN28A-mRNA networks that emerge in the different multiple trajectories arising during the reprogramming of mouse embryonic fibroblasts (MEFs) to iPSCs. The results suggest that LIN28A promotes specific cell identities in each of the productive or non-productive reprogramming trajectory branches, including the mesenchymal-to-epithelial transition (MET) (17). Our study sheds new light on LIN28A’s role in post-transcriptional regulation in the context of pluripotency and totipotency regulation *in vitro*, underscoring the relevance of understanding RNA-protein interactions in biological processes at the single-cell level.

## Results

### Identification of LIN28A mRNA targets in mouse ESCs at single-cell resolution

TRIBE involves fusing an RBP of interest to the catalytic domain of adenosine deaminase ADAR (ADARcd) and delivering this construct into target cells (18). This fusion protein allows the selective conversion of adenosines into inosines near the RNA region targeted by the specific RBP. The resulting modifications can be recognized as adenosine-to-guanine (A-to-G) edits during RNA-sequencing (RNA-seq). We engineered a fusion of LIN28A with ADARcd (LIN28A-ADARcd) and expressed it in E14 mouse ESCs for 24 h using a doxycycline-inducible construct. As expected, bulk RNA-seq showed a significant increase in the number and frequency of A-to-G mutation sites, without affecting the overall gene expression, when compared to cells expressing only ADARcd cells or wild-type (WT) (Fig. S1, *A*–*D*). This demonstrated that the induced A-to-G mutations are LIN28A-dependent, and the TRIBE system has no other obvious effects on mouse ESC function or characteristics.

**Figure 1:**
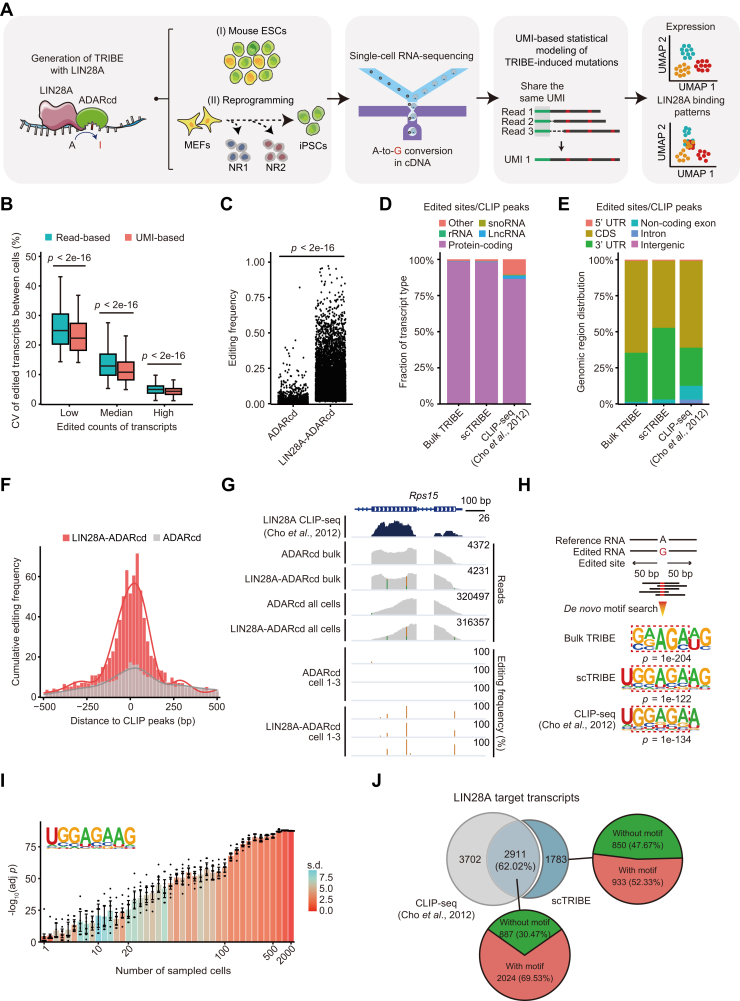
**Identification of LIN28A mRNA targets in mouse ESCs at single-cell resolution.***A*, schematic depicting the identification of LIN28A targets with scTRIBE in different pluripotency transition processes. NR, non-reprogramming. *B*, box plot showing the coefficient of variation (CV) percentages of read/UMI counts for edited transcripts between cells. Transcripts were categorized into three levels based on their average edited UMI counts: low (below the first quartile), median (between the first and third quartiles), and high (above the third quartile). *p* values were generated using a two-sided Wilcoxon test. *C*, dot plot showing the editing frequency in LIN28A-ADARcd and ADARcd control from scTRIBE results. Editing frequency was obtained by dividing the edited UMIs by the total UMIs per edited site. Only significantly edited sites in LIN28A-ADARcd are plotted. *p* values were generated using a two-sided Wilcoxon test. *D* and *E*, stacked bar plot showing the proportion of transcript types (*D*) and genomic region types (*E*) of the edited sites/CLIP peaks as identified by bulk TRIBE, scTRIBE, and CLIP-seq (11). snoRNA, small nucleolar RNA; rRNA, ribosomal RNA; lncRNA, long non-coding RNA; UTR, untranslated region; CDS, coding sequence. *F*, histogram and fitted curve plot showing the cumulative editing frequency distribution of LIN28A-ADARcd and ADARcd within a ± 500 bp window flanking the CLIP peaks (11). *G*, integrative genome viewer browser tracks showing the edited sites at the *Rps15* loci in bulk, pseudobulk, and single-cell level, all located within the LIN28A CLIP peaks (11). *H*, illustration of the top 1 enriched motif searched from the ± 50 bp region surrounding significantly edited sites of bulk and scTRIBE, or CLIP peaks (11). *I*, histogram showing the enrichment of the GGAGA-like motif using the ± 50 bp region surrounding significantly edited sites from randomly sampled cells. Data are the mean ± standard deviation (s.d.) of n = 10 computational trials. Adjusted *p* values (adj *p*) were generated using a binomial test and Benjamini-Hochberg correction. *J*, Venn diagram showing the overlap between targets identified by CLIP-seq (11) and scTRIBE (*top left*). Pie charts showing the proportion of transcripts that do not overlap (*top right*) or overlap (*bottom*) with CLIP-seq data and contain a GGAGA-like motifs within a ± 50 bp region surrounding the edited sites.

To identify the LIN28A targets at single-cell resolution, we applied scTRIBE to mouse E14 ESCs using the DNBelab C4 platform (Fig. 1*A*) (19). The dataset consisted of two sample types: LIN28A-ADARcd serving as the experimental group and ADARcd as the control group. The aggregated data included 11,121 cells, with an average of 65,578 reads per cell, a median gene count of 3756 per cell, and a median of 14,437 unique molecular identifiers (UMIs) per cell (Fig. S1*E*). Like in standard single-cell transcriptomic analysis, we observed that calibrating transcript editing levels using UMIs substantially reduces the coefficient of variation of LIN28A-ADARcd-induced mutations between cells (Fig. 1*B*). This indicated that UMIs offer a more precise metric than read counts for assessing RNA binding activity using scTRIBE. Therefore, all subsequent analyses in our study were conducted with UMIs. Next, we used a negative binomial distribution model to define edits specifically introduced by LIN28A-ADARcd (20). In total, we identified 11,631 edited sites across 4694 genes that exhibited a significantly higher editing frequency than the control (Fig. 1*C* and Table S1). We observed consistency in the abundance of transcript editing levels across replicates of the LIN28A-ADARcd samples, demonstrating good reproducibility (Fig. S1*F*).

To assess if the scTRIBE edits corresponded to LIN28A target RNAs, we conducted a comparative analysis between LIN28A-ADARcd edited transcripts and LIN28A CLIP-seq data from mouse ESCs (11). We observed that the RNA profiles from scTRIBE are similar to those from CLIP-seq. However, the scTRIBE result contained a higher proportion of protein-coding transcripts and decreased presence of non-coding RNAs (*e.g.,* snoRNA and rRNA), which is consistent with the nature of droplet-based scRNA-seq techniques that prefer sequencing 3′ ends of polyadenylated transcripts (Fig. 1*D*) (21). Besides, the genome-wide distribution of the edited sites in scTRIBE mirrored that of bulk TRIBE and CLIP-seq data, being predominantly localized within the coding sequence (CDS) and 3′ untranslated region (UTR) (Fig. 1*E*). These distributions are different from that of total reads and consistent with the known binding pattern of LIN28A (Fig. S1, *G* and *H*). Next, we measured the distance of the edited sites to the CLIP peaks, observing an enrichment of the scTRIBE edits within ± 250 bp of the LIN28A CLIP peaks (Fig. 1*F*). The tracks for the ribosomal factor *Rps15* are shown as an example (Fig. 1*G*). Moreover, *de novo* motif analysis of the edited sites revealed a significant presence of the canonical LIN28A binding motif (GGAGA-like sequence) in both the bulk and scTRIBE samples (Fig. 1*H*). This motif was also detected when downsizing from randomly sampled hundreds of cells to a single cell in the scTRIBE (Fig. 1*I*). Notably, 62.02% (2911) of the edited transcripts overlapped with CLIP-seq targets (Fig. 1*J*). Among the remaining 1783 non-overlapping transcripts, 933 (52.33%) contained GGAGA-like motifs located within ± 50 bp of the edited sites. The distinct targets identified by scTRIBE and CLIP-seq might arise from the variations of the cell lines (E14 *versus* A3-1 mouse ESCs), culture conditions (2i/LIF *versus* serum/LIF), or sequencing methods (bulk RNA-seq *versus* single-cell 3′ end RNA-seq) across the two studies.

These analyses demonstrate that scTRIBE recovers the mRNA targets of LIN28A within mouse ESCs at single-cell resolution.

### LIN28A has a unique mRNA-binding pattern in 2CLCs

**Figure 2:**
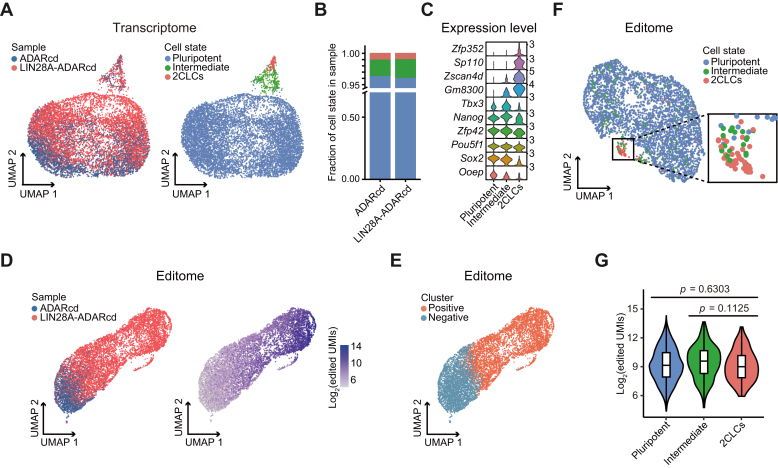
**LIN28A shows a unique mRNA-binding pattern in 2CLCs.***A*, UMAP plots showing the transcriptome of mouse ESCs from LIN28A-ADARcd and ADARcd control. The color denotes different samples (*left*) and cell states (*right*). *B*, stacked bar plot showing the proportion of cell states in LIN28A-ADARcd and ADARcd control. *C*, violin plot showing the expression of representative marker genes for different cell states. *D*, UMAP plots showing the editome of cells colored by sample (*left*) or number of edited UMIs (log_2_-transformed) (*right*). *E*, UMAP plot showing the editome of cells colored by cell cluster. *F*, UMAP plot showing the editome of LIN28A-ADARcd over-expressing cells identified in (*E*) colored by cell state. Zoom of the black-outlined box highlights the 2CLCs. *G*, violin plot showing the number of edited UMIs (log_2_-transformed) of LIN28A-ADARcd over-expressing cells in each cell state. *p* value was generated using a two-sided Wilcoxon test.

The mechanisms underlying 2CLC generation within mouse ESCs are not well understood (22). Although LIN28A controls this transition by repressing *Dux*, regulating the nucleolar structure, and promoting rRNA biosynthesis (8), it is unclear whether other mechanisms, specifically potential mRNA targets in 2CLCs, are involved. Because the 2CLC population within ESCs is, under normal conditions, small, this cannot be investigated with standard (bulk) CLIP-seq from cultured mouse ESCs. While it is possible to purify a sufficient number of 2CLCs, the process is lengthy and may affect the transcriptome. To study this at the single-cell level, we analyzed our scTRIBE experiments in mouse ESCs. We first combined the scRNA-seq datasets of LIN28A-ADARcd and ADARcd samples. Following Louvain clustering and Uniform Manifold Approximation and Projection (UMAP) visualization, we successfully categorized the cells into three distinct clusters (pluripotent ESCs, 96.1%; 2CLCs, 1.0%; intermediate pluripotent-2CLCs, or intermediate, 2.9%) using specific marker genes (Fig. 2, *A*–*C*). The observed proportions are consistent with those reported in the previous study (23). As expected, UMAP visualization of the editome (the collection of edited UMIs from each cell) revealed that cells expressing LIN28A-ADARcd could be distinguished from those expressing ADARcd (Fig. 2*D*). We also observed that a small subset of LIN28A-ADARcd cells had fewer edited UMIs, which could be due to insufficient fusion protein expression. To overcome this, we used Louvain clustering by editome and refined our datasets by excluding cells assigned to the cluster exhibiting fewer edited UMIs (Fig. 2*E*). The remaining cells from the LIN28A-ADARcd group were classified as the positive population (6212 cells) and were selected for further analysis. Within this positive population, 2CLCs were observed to form a distinct cluster based on the editome (Fig. 2*F*), despite having a similar edited UMI levels compared to the other two cell states (Fig. 2*G*). This indicated that LIN28A-bound RNA targets in 2CLCs are substantially different from those observed in pluripotent cells and intermediate cells.

### LIN28A interacts with ribosome biogenesis and totipotency factor mRNAs in 2CLCs

**Figure 3:**
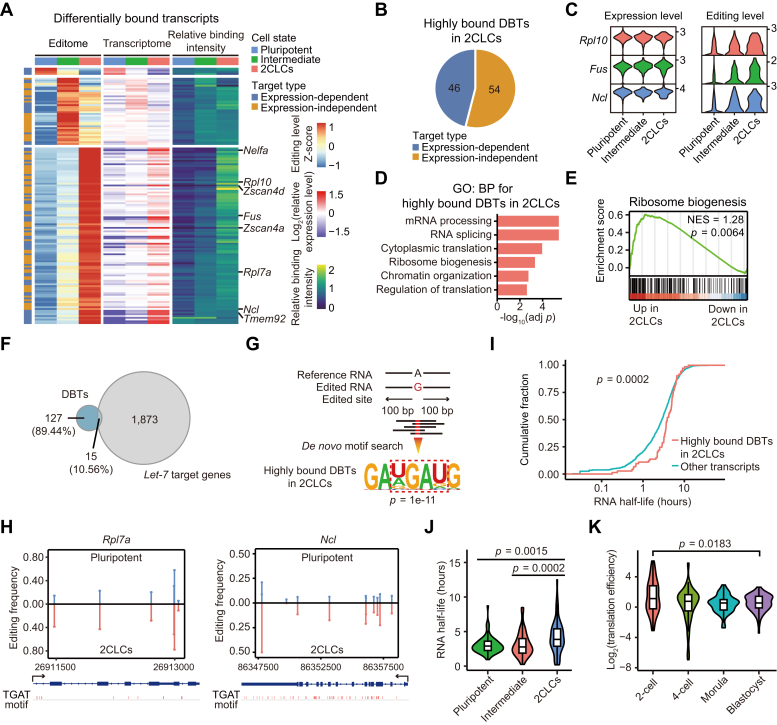
**LIN28A interacts with ribosome biogenesis and totipotency factor mRNAs in 2CLCs.***A*, clustered heatmaps showing the editing level (*left*), expression level (*middle*), and relative binding intensity (*right*) of 142 DBTs (fold-change > 1.2 with *p* value < 0.01) across three cell states. The relative binding intensity was calculated by dividing the relative editing level by the relative expression level. *p* value was generated using a two-sided Wilcoxon test. *B*, pie chart showing the proportion of expression-dependent and expression-independent LIN28A-highly bound DBTs in 2CLCs. The expression dependent-DBTs were determined as those with higher expression of the target gene (fold-change > 1.2 with *p* value < 0.01) in the corresponding cell type. *p* value was generated using a two-sided Wilcoxon test. *C*, violin plot showing the expression and editing levels of *Rpl10*, *Fus*, and *Ncl* in each cell state. *D*, bar plot showing the represented GO biological process terms of LIN28A-highly bound DBTs in 2CLCs. Adjusted *p* values were generated using a hypergeometric test and Benjamini-Hochberg correction. *E*, GSEA analysis showing the LIN28A binding intensity of transcripts related to ribosome biogenesis in 2CLCs compared with pluripotent cells. *p* value was generated using a permutation test. NES, normalized enrichment score. *F*, Venn diagram showing the overlap between transcripts differentially bound by LIN28A in all three cell states and *let-7* target genes. *G*, illustration of the top 1 enriched motif searched from the ± 100 bp region surrounding edited sites on LIN28A-highly bound DBTs in 2CLCs. *H*, editing frequency at each edited site on *Rpl7a* and *Ncl* in pluripotent cells and 2CLCs. UGAU motif (TGAT in DNA sequence) locations are shown. *I*, cumulative distribution plot showing the RNA half-lives (log_2_-transformed) of LIN28A-highly bound DBTs in 2CLCs and all other transcripts in single-cell RNA half-life dataset (23). *p* value was generated using a Kolmogorov-Smirnov test. *J*, violin plot showing the RNA half-lives of LIN28A-highly bound DBTs in 2CLCs across three cell states (23). *p* values were generated using a two-sided Wilcoxon test. *K*, violin plot showing the translation efficiency (log_2_-transformed) of LIN28A-highly bound DBTs in 2CLCs across different stages of the preimplantation embryo (33). *p* values were generated using a two-sided Wilcoxon test.

Next, we aimed to understand the differences in the scTRIBE editome between the three observed cell identities and what this could functionally mean for the 2CLCs. We performed differential editing analysis and identified 142 differentially bound transcripts (DBTs) (fold-change > 1.2 with *p* value < 0.01) among the three cell states (Fig. 3*A* and Table S2). 2CLCs contained the vast majority (100 out of 142) of the LIN28A-highly bound DBTs, with the intensity of binding defined as the ratio between editing level and expression level. These DBTs included transcripts belonging to genes highly expressed in the 2C-embryo and 2CLCs, such as the *Zscan4* family (*Zscan4a* and *Zscan4d*), *Tmem92,* and *Nelfa*. Additionally, we noticed that 54 of the LIN28A-highly bound DBTs in 2CLCs were not differentially expressed in 2CLCs compared to the other two cell states (Fig. 3*B*). These expression-independent DBTs could be related to protein and RNA modifications, and others (24, 25). Notably, genes such as *Rpl10*, *Fus*, and *Ncl*, which are intricately linked to nucleolus function (26, 27, 28), were included in this group (Fig. 3*C*). A smaller number of DBTs were found in the pluripotent (4 out of 142) and intermediate states (38 out of 142), respectively (Fig. 3*A*). Gene Ontology (GO) enrichment analysis of LIN28A-highly bound DBTs in 2CLCs confirmed the enrichment of factors involved in mRNA processing, cytoplasmic translation, and ribosome biogenesis (Fig. 3*D*). We also confirmed through gene set enrichment analysis (GSEA) that LIN28A-interacting ribosome biogenesis transcripts tend to have higher editing levels in 2CLCs compared to the pluripotent state (Fig. 3*E*). This observation was immediately interesting given the previous observations that LIN28A interacts with rRNAs, nucleolar factors, and ribosomal subunits in the 2C-embryo and 2CLCs, and that ribosomal proteins are key regulators of the 2C-embryo stage transcriptome (8, 29).

Given the classical role of LIN28A in inhibiting *let-7* miRNA maturation, we also compared genes differentially bound by LIN28A in all three cell states with previously reported *let-7* miRNA targets (Fig. 3*F*) (30). Remarkably, there was only a 10% gene overlap, suggesting that the identified DBTs in pluripotent cells and 2CLCs are largely independent of the *let-7* pathway and are likely directly regulated by LIN28A in a post-transcriptional manner. LIN28A is characterized by two distinct RNA-binding domains: a cold shock domain (CSD) and a pair of zinc finger-like CCHC domains. While both types of domains are crucial for inhibiting the maturation of *let-7* miRNA (31), the CSD domain is additionally related to the post-transcriptional processing of target mRNAs (32). We thus asked whether the observed DBTs in 2CLCs are predominantly recognized by the CSD domain. For this, we extracted the edited sites in these DBTs and performed *de novo* motif analysis (Fig. 3*G*). This showed enrichment of the UGAU-like motif, which is preferentially bound by the CSD domain (31), as the top 1 enriched motif. For instance, transcripts containing the UGAU-like sequence in *Rpl7a* and *Ncl* exhibited higher editing frequency in 2CLCs compared to pluripotent cells (Fig. 3*H*). Of note, previous studies have indicated that the CSD domain of LIN28A facilitates the expression of its target genes *via* enhancing mRNA stability (32). To address whether a similar regulation happens in 2CLCs, we analyzed a previously reported single-cell dataset of RNA stability in 2CLCs (23). Supporting our idea, we corroborated that LIN28A-highly bound transcripts have significantly longer half-lives compared to other transcripts in 2CLCs (Fig. 3*I*). Moreover, these transcripts also showed longer half-lives in 2CLCs than in the other two cell states (Fig. 3*J*). Besides, by analyzing an mRNA translation dataset of mouse developing embryos (33), these transcripts also exhibited higher translation efficiency in mouse 2C-embryo than in other preimplantation embryo stages (Fig. 3*K*).

These findings suggest that LIN28A contributes to making a subset of highly bound genes in 2CLCs more stable and/or more translated, potentially through the regulatory role of the CSD domain. Further experiments will be necessary to dissect the different regulators that act coordinately with the CSD domain to stabilize LIN28A mRNA targets.

### Dynamic LIN28A-mRNA interactions during somatic cell reprogramming

LIN28A was one of the original exogenous factors enabling human somatic cell reprogramming into iPSCs (34). LIN28A also promotes somatic cell reprogramming in mice (35). It is known that LIN28A plays a crucial role in metabolic remodeling and proliferation during this process (36, 37), but the underlying mechanisms are unclear. To explore this, we first analyzed the expression of endogenous *Lin28a* during the reprogramming of MEFs into iPSCs under the highly efficient iCD1 culture medium (38). The use of this medium was important to reduce variability and shorten the duration of the reprogramming process. Reverse transcription-quantitative PCR (RT-qPCR) showed that *Lin28a* increased with reprogramming reaching a peak in the later stages (Fig. S2*A*). Moreover, knocking it down impaired cell proliferation, a major feature of reprogramming (36), and reduced reprogramming efficiency (Fig. S2, *B*–*D*).

**Figure 4:**
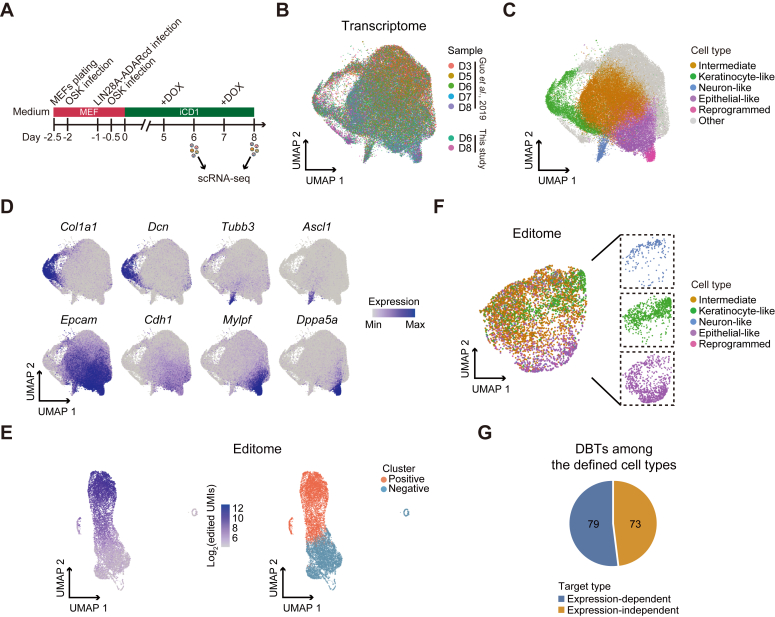
**LIN28A has different mRNA-binding patterns across somatic cell reprogramming.***A*, schematic showing the application of scTRIBE to iCD1-OSK reprogramming. *B*, UMAP plot showing the transcriptome of integrated cells of reprogramming spanning from day 3 (D3) to day 8 (D8) from published data (39) and this study. Cells are colored by sample. *C*, UMAP plot showing cells colored by cell type. *D*, UMAP plots showing the expression levels of representative marker genes for different cell types. *E*, UMAP plots showing the editome of cells from this study. Cells are colored by the number of edited UMIs (log_2_-transformed) (*left*) and cell cluster (*right*). *F*, UMAP plot showing the editome of defined cell types (*left*). Split UMAP plots highlight distinct cell types (*right*). *G*, pie chart showing the proportion of expression-dependent and expression-independent DBTs among the defined cell types. DOX, doxycycline.

Next, we applied scTRIBE to investigate the dynamic interactions between LIN28A and its target mRNAs in iCD1-mediated reprogramming (Fig. 4*A*). Given that *Lin28a* expression becomes higher in the mid-late stages, we induced LIN28A-ADARcd expression with doxycycline on day 5 (D5) and day 7 (D7), followed by cell collection for scRNA-seq 24 h later. LIN28A-ADARcd expression was confirmed by western blotting (Fig. S2*E*). After quality control, the generated data encompassed a total of 16,337 cells, with an average of 23,438 reads per cell, a median gene count of 3814 per cell, and a median of 10,426 UMIs per cell (Fig. S2*F*). To study the role of LIN28A throughout the reprogramming trajectories, we integrated our own scRNA-seq dataset with published scRNA-seq data using the same reprogramming system from day 3 (D3) until day 8 (D8) (39), observing a good match (Figs. 4*B* and S2*G*). Cells from different branches of the reprogramming process were identified in this analysis, including fully reprogrammed cells, epithelial-like cells, neuronal-like cells, and keratinocyte-like cells, each characterized by unique marker genes (39), the cluster beginning to express epithelial genes was defined as being in an intermediate state, consistent with previous knowledge that the MET is an essential step of reprogramming (Fig. 4, *C* and *D*) (17). After excluding cells with insufficient edits, we identified 5731 positive cells in our scRNA-seq dataset (Fig. 4*E*). These cells mostly exhibited similar editing levels when categorized by time points or cell types (Fig. S2, *H* and *I*). We also observed that fully reprogrammed cells displayed lower levels of editing, which is likely due to the silencing of viral vectors after completing reprogramming (40). Notably, epithelial-like, keratinocyte-like, and neuronal-like cells formed distinct clusters in the editome-based UMAP analysis (Fig. 4*F*). Among the defined cell types, we observed 152 DBTs (fold-change > 1.2 with *p* value < 0.01), 79 of which were gene expression-dependent while 73 were not (Fig. 4*G*).

**Figure 5:**
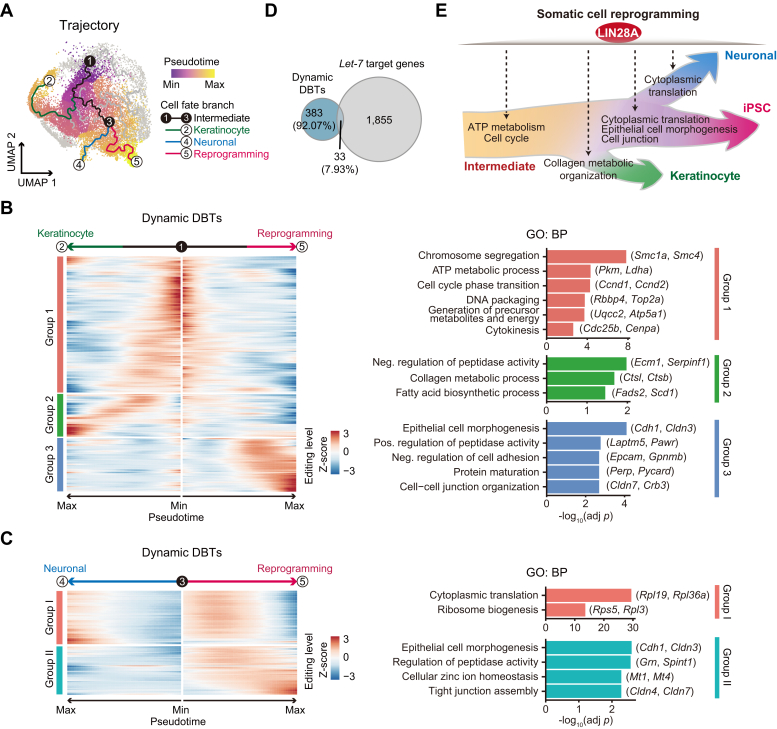
**LIN28A-mRNA binding dynamics across the different trajectories of somatic cell reprogramming.***A*, UMAP plot showing the reprogramming trajectories and cells colored by pseudotime. Four cell fate branches are highlighted: intermediate branch (*black*), keratinocyte branch (*green*), neuronal branch (*blue*), and successful reprogramming branch (*magenta*). The *black* nodes indicate the start and end point of the intermediate branch, and the white nodes represent the endpoints of the remained three cell fate branches. Only cells from this study are shown. *B*, heatmap showing the editing levels of 342 dynamic DBTs (cataloged in three groups, *q* value < 0.01) between keratinocyte and successful reprogramming trajectories in a pseudotime order (*left*). *q* values were generated using a likelihood ratio test. Bar plots showing the enriched GO biological process terms and representative transcripts (*right*). Adjusted *p* values were generated using a hypergeometric test and Benjamini-Hochberg correction. Neg., negative; Pos., positive. *C*, heatmap showing the editing levels of 143 dynamic DBTs (cataloged in two groups) between neuronal and successful reprogramming trajectories in a pseudotime order (*left*). Bar plots showing the enriched GO biological process terms and representative transcripts (*right*). Adjusted *p* values were generated using hypergeometric test and Benjamini-Hochberg correction. *D*, venn diagram showing the overlap between dynamic DBTs identified in all cell fate branches and *let-7* target genes. *E*, schematic model showing the dynamic LIN28A-mRNA interactions in different cell fate branches during somatic cell reprogramming.

Next, we used Monocle3 (41), placing D3 cells at the beginning of a pseudotime to identify the major trajectories across the reprogramming process. This showed several branch points including an intermediate branch, a failed keratinocyte branch, a failed neuronal branch, and a branch representing successful reprogramming at the endpoint of the pseudotime (Fig. 5*A*). We first focused on the cells in the keratinocyte and successful reprogramming trajectories, identifying 342 edited transcripts (*q* value < 0.01) that dynamically interacted with LIN28A (hereafter referred to as dynamic DBTs) along the entire pseudotime. These transcripts exhibited three major binding patterns (Fig. 5*B* and Table S3). Group 1 represented transcripts with high editing levels in the early-mid stage of the intermediate branch, comprising genes related to chromosome segregation (*Smc1a*, *Smc4*), cell cycle phase transition (*Ccnd1*, *Ccnd2*), and ATP metabolic process (*Pkm*, *Ldha*). Group 2 represented transcripts with a gradually increased editing levels towards the failed branch, including genes related to negative regulation of peptidase activity (*Ecm1*, *Serpinf1*) and collagen metabolic organization (*Ctsl*, *Ctsb*). Group 3 represented transcripts with increased editing towards the successful reprogramming branch, comprising genes related to epithelial morphogenesis (*Cdh1*, *Cldn3*) and cell-cell junction organization (*Cldn7*, *Crb3*), suggesting that LIN28A may play a crucial role in the MET process.

Interestingly, LIN28A has also been shown to regulate neuronal and axon regeneration (42, 43), so we looked into the neuronal-like trajectory. Compared with the successful reprogramming, we identified 143 dynamic DBTs, exhibiting two major patterns (Fig. 5*C* and Table S3). Group I was more enriched in the later phase of neuronal cell fate acquisition and corresponded to genes related to ribosome biogenesis (*Rpl19*, *Rpl36a*) and protein translation (*Rps5*, *Rpl3*), which to a lesser extent were enriched in the middle phase of the successful reprogramming trajectory. Group II contained transcripts with high editing levels in the reprogramming trajectory and low in the neuronal-like one. It has been reported that LIN28A modulates neuronal regeneration by inducing the mTOR translation activation pathway (43). We compared the LIN28A interacting dynamically bound genes in these two major trajectories with *let-7* miRNA targets (30), observing only 7.93% overlap (Fig. 5*D*). We thus concluded that LIN28A dynamic DBTs in reprogramming are mainly regulated in a *let-7*-independent manner.

Our results demonstrate that LIN28A has a variety of mRNA targets and functions across the somatic cell reprogramming process (Fig. 5*E*), helping promote individual cell identities and blocking others along the dynamic conversions.

## Discussion

We have applied scTRIBE to dissect the mRNA targets of LIN28A in mouse ESCs, 2CLCs, and somatic cell reprogramming. This method distinguishes itself from traditional techniques like CLIP-seq by enabling the detection of RNA-protein interactions at the single-cell level. Although CLIP-seq had been previously used to profile LIN28A RNA targets in mouse ESCs (11), it does not consider the heterogeneity of ESC cultures and, hence, cannot be used for the detection of rare populations such as 2CLCs within mouse ESCs. Likewise, despite being a well-known regulator of somatic cell reprogramming, the RNA targets of LIN28A in this multi-path process are yet unclear, and applying CLIP-seq to bulk mixed populations would provide limited information.

LIN28A is known to promote rRNA maturation and maintain nucleolar integrity in the 2C-embryo and 2CLCs (8). Interestingly, our analysis showed that transcripts highly bound by LIN28A in 2CLCs include ribosome biogenesis factors and a selected panel of totipotent factors such as members of the *Zscan4* family of transcription factors. These totipotency factors are not expressed in ESCs. Considering the role of LIN28A in promoting mRNA stability and translation (32), it is plausible to think that in 2CLCs they are regulated by LIN28A in this same manner. As for the ribosome biogenesis factors targeted by LIN28A in 2CLCs, their translation is maintained at high levels in the 2C-embryo, also suggesting that the same occurs in 2CLCs. In fact, we also observed that despite the mRNA levels of these factors is similar compared to ESCs, their mRNA half-life is substantially increased. This suggests a compensatory mechanism for counterbalancing the more general suppression of translation mediated by repressing rRNA transcription and the partial dismantling of nucleolar structure executed by LIN28A in the totipotent state (8). Such a mechanism could facilitate that the expression of totipotency-related factors is selectively maintained despite the overall translation efficiency being reduced. Further experimentation will be necessary to understand the cause-effect relationship between these observations.

In somatic cell reprogramming, we observed that LIN28A interacts with mRNAs associated with metabolic processes and cell cycle remodeling in the early phase of reprogramming before cells branch into the productive and unproductive reprogramming phases. This is consistent with previous observations describing a role for LIN28A in these processes during reprogramming (36, 37). LIN28A also binds to transcripts related to epithelial morphogenesis in the middle phase of reprogramming, indicating a facilitating effect on the MET (17). Besides, we have demonstrated that LIN28A interacts with transcripts related to cytosolic translation in neuronal fate. In this regard, it is known that LIN28A modulates axon regeneration in neurons by inducing the mTOR translation activation pathway (43). Notably, these mRNA targets are independent of *let-7*, a miRNA with pleiotropic functions that is a well-characterized target of LIN28A (44), as demonstrated by performing overlap analysis. These findings support a model in which LIN28A reinforces different cell fates by directly promoting the expression of key cell-specific factors and/or regulating the translational machinery.

Our work has certain limitations. For example, owing to the inherent constraints of droplet-based scRNA-seq, which primarily targets 3′ end sequences (45), there is a reduction in the proportion of 5′ UTR- and CDS-edited sites in the scTRIBE results. Using full-length RNA coverage methods such as Smart-seq and VASA-seq may help solve this issue (46, 47). Moreover, the current version of scTRIBE relies on exogenously expressed fusion proteins. Developing methods based on endogenous proteins could provide more accurate insights into RBP-RNA interactions. Nevertheless, our dataset is a useful resource for understanding how LIN28A regulates cell fate transitions including the pluripotent-to-totipotent and somatic cell reprogramming transitions. In the future, we envisage that the application of refined scTRIBE approaches based on further advanced technologies such as long-read sequencing will help create comprehensive maps of protein-RNA interactions with isoform resolution in a variety of cell contexts and situations, greatly enhancing our understanding of those processes (48).

## Experimental procedures

### Plasmid and virus preparation

LIN28A-ADARcd construct was engineered by fusing the mouse *Lin28a* coding sequence with the mouse *Adar2* catalytic domain containing a hyperactive mutant E488Q (hereafter referred to as ADARcd). ADARcd and LIN28A-ADARcd constructs used for mouse ESCs were subcloned into a doxycycline-inducible piggyBac vector with a FLAG tag. The LIN28A-ADARcd construct used for reprogramming experiments was subcloned into a doxycycline-inducible pW lentiviral vector. pMXs vectors, which independently express the *Oct4*, *Sox2,* and *Klf4* (OSK), were purchased from Addgene. shRNA oligonucleotides targeting *Lin28a* were subcloned into pLKO.1 lentiviral vector. Detailed sequences of these constructs are provided in Table S4.

To obtain the lentiviruses and retroviruses used in reprogramming experiments, HEK293T cells at 70 to 80% confluence were transduced with the pW vector carrying LIN28A-ADARcd or the pLKO.1 vector carrying shRNA oligos, along with packaging vectors psPAX2 and pMD2G using polyethyleneimine (PEI; Polysciences) for 8 h. Plat-E cells at 70 to 80% confluence were transduced with retroviral vectors containing OSK, or rtTA using PEI for 8 h 48 h later after transduction, the viruses were harvested and filtered through a 0.45 μm filter (Millipore).

### Cell culture

OG2 MEFs carry multiple copies of the *Oct4*-GFP transgenic reporter. HEK293T, Plat-E cells and OG2 MEFs were maintained in Dulbecco’s modified Eagle’s medium (DMEM)/high glucose (Corning) supplemented with 10% fetal bovine serum (FBS; NATOCOR), GlutaMAX (Gibco), nonessential amino acids (NEAA; BasalMedia), and penicillin/streptomycin (P/S; HyClone). E14gt2a (E14) ESCs were cultured in 2i/LIF medium containing DMEM/F12 (HyClone) and Neurobasal (Gibco) mixed 1:1, supplemented with N2 (Gibco), B27 (Gibco), GlutaMAX, NEAA, sodium pyruvate, β-mercaptoethanol, P/S, leukemia inhibitory factor (1000 U/ml), 3 μM CHIR99021 (StemRD) and 1 μM PD0325901 (StemRD). Cells were grown in 5% CO_2_ at 37 °C and the medium was changed daily. All cells tested negative for *mycoplasma*.

### Application of TRIBE to mouse ESCs

To establish a stable TRIBE system, E14 mouse ESCs were plated at 80,000 cells per well in a 6-well plate and transfected with piggyBac vector containing ADARcd/LIN28A-ADARcd and the transposase vector pBase using Lipofectamine 3000 (Invitrogen) following the manufacturer’s instruction. 48 h after transduction, the mouse ESCs were selected using 1 μg/ml puromycin (InvivoGen) for 4 days. The generated cell lines expressing ADARcd and LIN28A-ADARcd were induced with 1 μg/ml doxycycline (DOX) for 24 h. Subsequently, these cells were harvested for either bulk or scRNA-seq.

### Application of TRIBE to somatic cell reprogramming

Somatic cell reprogramming was performed as previously described (39). Briefly, OG2 MEFs at passage two were seeded at a density of 20,000 cells per well in 12-well plates and then infected with the virus after 12 h. OG2 MEFs were first infected with OSK retroviruses for 24 h and then infected with inducible LIN28A-ADARcd lentivirus for 6 h, and finally infected with OSK and rtTA retroviruses for 12 h. After infection, the cells were cultured in iCD1 medium and we designated this time point as day 0 (38). Next, cells were induced with 1 μg/ml DOX in iCD1 medium for 24 h on D5 and D7. The collected cells were thus marked as D6 and D8 and harvested for scRNA-seq. For LIN28A knockdown experiments, shRNA lentiviruses were diluted at 1:30 with fresh medium, and 1 round of 8 h of infection was performed between the retrovirus infections. *Oct4*-GFP positive colonies were scanned by Sapphire Biomolecular Imager (Azure Biosystems) on D8 and counted using the *Analyze Particles* function in ImageJ (v.1.50i).

### Western blotting

Cells were lysed and subjected to SDS-PAGE gel for separation and transferred onto a PVDF membrane (Millipore). The membrane was subsequently blocked and incubated with corresponding primary and secondary antibodies. The signal was generated with ECL (Advansta) and visualized with a FUSION SOLO 4M System (Vilber Lourmat). Primary antibodies used in this paper are as follows: anti-FLAG (Sigma-Aldrich F7425), anti-LIN28A (Abcam ab46020), anti-Histone H3 (Abcam ab1791), and anti-ACTIN (Sigma-Aldrich A2066).

### RNA isolation, RT-qPCR, and bulk RNA-seq library preparation

Total RNA samples were extracted with TRIzol (MRC). RT-qPCR was performed using SYBR Green (Roche) with a Roche LightCycler 96 instrument. Primer sequences are provided in the Table S4. RNA-seq libraries were constructed using the ribosomal RNA deletion method and then sequenced on the DIPSEQ T1 platform, generating ∼30 million 100 bp paired-end reads per sample.

### scRNA-seq library construction

scRNA-seq library preparation was performed using the DNBelab C Series Single-Cell Library Prep Kit (MGI) according to the manufacturer’s instructions. Briefly, single-cell suspensions were used for droplet generation, emulsion breakage, mRNA-captured bead collection, reverse transcription, second-strand synthesis, cDNA amplification, and purification to generate barcoded libraries. The sequencing libraries were assessed using an Agilent Bioanalyzer and sequenced on the DIPSEQ T1 platform.

### RNA editome analysis in bulk-level TRIBE

Raw data were first trimmed of adapters and filtered out low-quality reads using fastp (v.0.20.1). Cleaned reads were then aligned to the mm10 reference genome using HISAT2 (v.2.2.1). Next, the aligned reads in SAM format were converted to BAM format, with the removal of reads having a mapping quality score < 10, followed by the depletion of PCR duplicates using sambamba (v.0.8.8). Finally, the number of aligned reads mapped to GENCODE annotations (v.M24) were counted using Subread featureCounts (v.2.0.2). The processed BAM files were subjected to mutation calling using bcftools (v1.10.2) with mm10 reference genome, the identified mutations in the dbSNP database (GCA_000001635.6_current_ids.vcf.gz) were removed. Next, we analyzed the mutations from annotated mRNA transcripts using the in-house workflow TRIBE (http://github.com/shiquan/scTRIBE). Briefly, we mapped the mutation sites onto the transcripts and conducted strand-specific calibration, which included A-to-G mutations on the sense strand and T-to-C mutations on the antisense strand. We then used beta-binomial distribution to model the RNA editing frequencies as previously described (20). Significantly edited sites were determined by (1) false discovery rate (FDR) < 0.01, (2) editing frequency between 0.05 and 0.95 in each replicate, (3) number of edited reads > 2 in each replicate, and (4) coverage of edited sites > 10 in each replicate.

### CLIP-seq analysis

The CLIP-seq dataset (11) was processed following the trimming, aligning, quality-checking, and PCR duplicates removing procedure as in the ‘RNA editome analysis in bulk-level TRIBE’ described earlier. Next, we used CLIPer (v.2.1.2, https://github.com/YeoLab/clipper) to call the significantly enriched peaks, the peaks with *p* value < 0.01 and the number of reads within the peaks > 6 in each replicate were kept for further analysis. Finally, the peaks were annotated against GENCODE annotations (v.M24).

### Motif analysis

For the *de novo* motif that surrounds LIN28A-ADARcd edited sites from bulk RNA-seq and pseudo-bulk scRNA-seq datasets, we used *findMotifsGenome.pl* function in Homer (v.4.9.1) with the parameter ‘mm10r -size 100 -rna -len 7,8’. To assess the enrichment of the GGAGA-like motif surrounding the edited sites from randomly sampled cells and from genes that non-overlapping with CLIP-seq, we performed *findMotifsGenome.pl* with the parameter ‘mm10r -mknown GGAGA-like.motif -nomotif -size 100 -norevopp’. For *de novo* motif finding surrounding the edited sites on highly bound DBTs in 2CLCs, we performed *findMotifsGenome.pl* with the parameter ‘mm10 -size 200 -rna -len 7’.

### scRNA-seq transcriptome analysis

Raw sequencing reads were filtered, demultiplexed, and aligned to the mm10 reference genome using an in-house workflow (https://github.com/MGI-tech-bioinformatics/DNBelab_C_Series_HT_scRNA-analysis-software/). The resulting transcriptome matrices were loaded into the Seurat package (v.4.0.4) in R (v.4.1.0) for downstream analysis unless otherwise specified. Cells with fewer than 2000 or more than 100,000 detected UMIs or over 10% of mitochondrial genes were removed. Doublets were filtered using DoubletFinder (v.2.0.3). To integrate reprogramming scRNA-seq data from Lin Guo *et al.* (39) with our dataset, the *FindIntegrationAnchors* function was used followed by the *IntegrateData* with default parameters. All data were normalized and the top 2000 highly variable genes were identified and scaled with regressing out cell cycle genes. Principal component analysis was performed and the first ten principal components (PCs) (mouse ESCs) or 20 PCs (reprogramming) were used for UMAP reduction. Clusters were identified using Louvain at resolution 2. High expression genes were identified using the *FindAllMarkers* with a two-sided Wilcoxon rank-sum test (*p* value < 0.01) and the logfc.threshold parameter set to 0.263 (fold-change > 1.2). To identify major cell types, adjacent clusters with similar marker gene expression were combined to a single cell cluster. In reprogramming, a small cell cluster (1.76% of input cells) was identified as ‘cells with stress and apoptosis’ based on previously reported markers (*Nupr1*, *Ddit3*, *Hspa5*, *Ctsd*, *Cebpb,* and *Atf3*) (13) and was excluded. For pseudotime analysis, cells of D3 and D5 from Lin Guo *et al.*, D6 and D8 from this study were loaded into the monocle3 package (v.1.0.0). Cells were clustered followed by trajectory learning without closing loop, and then ordered in a pseudotime using D3 as the root of the trajectory.

### RNA editome analysis in scTRIBE

The processed BAM files of scRNA-seq were used for mutation calling as in the ‘RNA editome analysis in bulk-level TRIBE’ described above, except that at the single-cell level, we used the number of UMIs instead of reads at each edited site. The single-cell editome matrices were then constructed by aggregating UMI counts of the significantly edited sites located in the exon of each gene and loaded into the Seurat for downstream analysis unless otherwise specified. The cells with fewer than two edited transcripts or 20 edited UMIs were discarded. To quantify the binding intensity of the LIN28A, edited UMIs of genes were divided by the total UMIs of that cell, multiplied by 10,000, and log-transformed. The top 600 highly variable genes were scaled and subjected to principal component analysis. The first 8 PCs were selected and used for UMAP reduction. Clusters were identified using Louvain at resolution 0.1. The cells assigned to the cluster that did not contain substantial editing were defined as negative population, and remained cells were defined as positive population and used for further analysis. DBTs were identified using *FindAllMarkers* with a two-sided Wilcoxon rank-sum test (*p* value < 0.01) and the logfc.threshold parameter set to 0.263 (fold-change > 1.2). The expression dependent-DBTs were determined as those with higher expression of the target gene (fold-change > 1.2 with *p* value < 0.01). in the corresponding cell type. GO enrichment and GSEA analysis were performed using *enrichGO* and *gseGO* in the clusterProfiler package (v.4.0.5), respectively. For mouse ESCs, the average editing and expression levels of DBTs in each cell state were calculated using *AverageExpression*. Single-cell RNA stability dataset was collected from Qiu *et al.* (23) to compare the RNA half-lives of highly bound DBTs in the 2CLCs with other detected genes, and RNA half-lives of the same DBTs across three different cell states. mRNA translation dataset was collected from Zhang *et al.* (33) to compare the translation efficiency of highly bound DBTs in 2CLCs across different stages of mouse preimplantation development. For reprogramming, cell data of different trajectories were subset and then loaded into the monocle package (v.2.22.0). Dynamic DBTs were identified using the *differentialGeneTest* with fullModelFormulaStr parameter set to ‘∼sm.ns (Pseudotime)’ and only the genes with *q* value < 0.01 were selected for further analysis. Heatmaps were plotted based on the *genSmoothCurves* function in the monocle.

## Data availability

The sequencing data generated in this study have been deposited on the CNGB Sequence Archive (CNSA) of China National GeneBank DataBase (CNGBdb) with accession number CNP0005202. All other data are present within the manuscript and supporting information.

## Supporting information

This article contains supporting information.

## Conflict of interest

The authors declare that they have no conflicts of interest with the contents of this article.

## References

[1] Li M., Belmonte J.C. (2017). Ground rules of the pluripotency gene regulatory network. Nat. Rev. Genet..

[2] Hu Y., Yang Y., Tan P., Zhang Y., Han M., Yu J. (2023). Induction of mouse totipotent stem cells by a defined chemical cocktail. Nature.

[3] Ye J., Blelloch R. (2014). Regulation of pluripotency by RNA binding proteins. Cell Stem Cell.

[4] Corley M., Burns M.C., Yeo G.W. (2020). How RNA-binding proteins interact with RNA: molecules and mechanisms. Mol. Cell.

[5] Hentze M.W., Castello A., Schwarzl T., Preiss T. (2018). A brave new world of RNA-binding proteins. Nat. Rev. Mol. Cell Biol..

[6] Shyh-Chang N., Daley G.Q. (2013). Lin28: primal regulator of growth and metabolism in stem cells. Cell Stem Cell.

[7] Hafner M., Max K.E., Bandaru P., Morozov P., Gerstberger S., Brown M. (2013). Identification of mRNAs bound and regulated by human LIN28 proteins and molecular requirements for RNA recognition. RNA.

[8] Sun Z., Yu H., Zhao J., Tan T., Pan H., Zhu Y. (2022). LIN28 coordinately promotes nucleolar/ribosomal functions and represses the 2C-like transcriptional program in pluripotent stem cells. Protein Cell.

[9] Ramanathan M., Porter D.F., Khavari P.A. (2019). Methods to study RNA-protein interactions. Nat. Methods.

[10] Lee F.C.Y., Ule J. (2018). Advances in CLIP technologies for studies of protein-RNA interactions. Mol. Cell.

[11] Cho J., Chang H., Kwon S.C., Kim B., Kim Y., Choe J. (2012). LIN28A is a suppressor of ER-associated translation in embryonic stem cells. Cell.

[12] Kolodziejczyk A.A., Kim J.K., Tsang J.C., Ilicic T., Henriksson J., Natarajan K.N. (2015). Single cell RNA-sequencing of pluripotent states unlocks modular transcriptional variation. Cell Stem Cell.

[13] Schiebinger G., Shu J., Tabaka M., Cleary B., Subramanian V., Solomon A. (2019). Optimal-transport analysis of single-cell gene expression identifies developmental trajectories in reprogramming. Cell.

[14] McMahon A.C., Rahman R., Jin H., Shen J.L., Fieldsend A., Luo W. (2016). TRIBE: hijacking an RNA-editing enzyme to identify cell-specific targets of RNA-binding Proteins. Cell.

[15] Brannan K.W., Chaim I.A., Marina R.J., Yee B.A., Kofman E.R., Lorenz D.A. (2021). Robust single-cell discovery of RNA targets of RNA-binding proteins and ribosomes. Nat. Methods.

[16] Sekar V., Marmol-Sanchez E., Kalogeropoulos P., Stanicek L., Sagredo E.A., Widmark A. (2023). Detection of transcriptome-wide microRNA-target interactions in single cells with agoTRIBE. Nat. Biotechnol..

[17] Li R., Liang J., Ni S., Zhou T., Qing X., Li H. (2010). A mesenchymal-to-epithelial transition initiates and is required for the nuclear reprogramming of mouse fibroblasts. Cell Stem Cell.

[18] Rahman R., Xu W., Jin H., Rosbash M. (2018). Identification of RNA-binding protein targets with HyperTRIBE. Nat. Protoc..

[19] Han L., Wei X., Liu C., Volpe G., Zhuang Z., Zou X. (2022). Cell transcriptomic atlas of the non-human primate *Macaca fascicularis*. Nature.

[20] Nguyen D.T.T., Lu Y., Chu K.L., Yang X., Park S.M., Choo Z.N. (2020). HyperTRIBE uncovers increased MUSASHI-2 RNA binding activity and differential regulation in leukemic stem cells. Nat. Commun..

[21] Zhang X., Li T., Liu F., Chen Y., Yao J., Li Z. (2019). Comparative analysis of droplet-based ultra-high-throughput single-cell RNA-seq systems. Mol. Cell.

[22] Genet M., Torres-Padilla M.E. (2020). The molecular and cellular features of 2-cell-like cells: a reference guide. Development.

[23] Qiu Q., Hu P., Qiu X., Govek K.W., Camara P.G., Wu H. (2020). Massively parallel and time-resolved RNA sequencing in single cells with scNT-seq. Nat. Methods.

[24] Sternburg E.L., Gruijs da Silva L.A., Dormann D. (2022). Post-translational modifications on RNA-binding proteins: accelerators, brakes, or passengers in neurodegeneration?. Trends Biochem. Sci..

[25] Lewis C.J.T., Pan T., Kalsotra A. (2017). RNA modifications and structures cooperate to guide RNA–protein interactions. Nat. Rev. Mol. Cell Biol..

[26] Dorner K., Ruggeri C., Zemp I., Kutay U. (2023). Ribosome biogenesis factors-from names to functions. EMBO J..

[27] Gawade K., Plewka P., Hafner S.J., Lund A.H., Marchand V., Motorin Y. (2023). FUS regulates a subset of snoRNA expression and modulates the level of rRNA modifications. Sci. Rep..

[28] Yu H., Sun Z., Tan T., Pan H., Zhao J., Zhang L. (2021). rRNA biogenesis regulates mouse 2C-like state by 3D structure reorganization of peri-nucleolar heterochromatin. Nat. Commun..

[29] Yi Y., Zeng Y., Sam T.W., Hamashima K., Tan R.J.R., Warrier T. (2023). Ribosomal proteins regulate 2-cell-stage transcriptome in mouse embryonic stem cells. Stem Cell Rep..

[30] Chen Y., Wang X. (2020). miRDB: an online database for prediction of functional microRNA targets. Nucleic Acids Res..

[31] Ustianenko D., Chiu H.S., Treiber T., Weyn-Vanhentenryck S.M., Treiber N., Meister G. (2018). LIN28 selectively modulates a subclass of let-7 MicroRNAs. Mol. Cell.

[32] Yamamoto H., Uchida Y., Kurimoto R., Chiba T., Matsushima T., Ito Y. (2023). RNA-binding protein LIN28A upregulates transcription factor HIF1alpha by posttranscriptional regulation via direct binding to UGAU motifs. J. Biol. Chem..

[33] Zhang C., Wang M., Li Y., Zhang Y. (2022). Profiling and functional characterization of maternal mRNA translation during mouse maternal-to-zygotic transition. Sci. Adv..

[34] Yu J., Vodyanik M.A., Smuga-Otto K., Antosiewicz-Bourget J., Frane J.L., Tian S. (2007). Induced pluripotent stem cell lines derived from human somatic cells. Science.

[35] Buganim Y., Markoulaki S., van Wietmarschen N., Hoke H., Wu T., Ganz K. (2014). The developmental potential of iPSCs is greatly influenced by reprogramming factor selection. Cell Stem Cell.

[36] Hanna J., Saha K., Pando B., van Zon J., Lengner C.J., Creyghton M.P. (2009). Direct cell reprogramming is a stochastic process amenable to acceleration. Nature.

[37] Zhang J., Ratanasirintrawoot S., Chandrasekaran S., Wu Z., Ficarro S.B., Yu C. (2016). LIN28 regulates stem cell metabolism and conversion to primed pluripotency. Cell Stem Cell.

[38] Chen J., Liu J., Chen Y., Yang J., Chen J., Liu H. (2011). Rational optimization of reprogramming culture conditions for the generation of induced pluripotent stem cells with ultra-high efficiency and fast kinetics. Cell Res..

[39] Guo L., Lin L., Wang X., Gao M., Cao S., Mai Y. (2019). Resolving cell fate decisions during somatic cell reprogramming by single-cell RNA-seq. Mol. Cell.

[40] Hotta A., Ellis J. (2008). Retroviral vector silencing during iPS cell induction: an epigenetic beacon that signals distinct pluripotent states. J. Cell Biochem..

[41] Cao J., Spielmann M., Qiu X., Huang X., Ibrahim D.M., Hill A.J. (2019). The single-cell transcriptional landscape of mammalian organogenesis. Nature.

[42] Wang X.W., Li Q., Liu C.M., Hall P.A., Jiang J.J., Katchis C.D. (2018). Lin28 signaling supports mammalian PNS and CNS axon regeneration. Cell Rep..

[43] Nathan F.M., Ohtake Y., Wang S., Jiang X., Sami A., Guo H. (2020). Upregulating Lin28a promotes axon regeneration in adult mice with optic nerve and spinal cord Injury. Mol. Ther..

[44] Viswanathan S.R., Daley G.Q. (2010). Lin28: a microRNA regulator with a macro role. Cell.

[45] Ding J., Adiconis X., Simmons S.K., Kowalczyk M.S., Hession C.C., Marjanovic N.D. (2020). Systematic comparison of single-cell and single-nucleus RNA-sequencing methods. Nat. Biotechnol..

[46] Picelli S., Bjorklund A.K., Faridani O.R., Sagasser S., Winberg G., Sandberg R. (2013). Smart-seq2 for sensitive full-length transcriptome profiling in single cells. Nat. Methods.

[47] Salmen F., De Jonghe J., Kaminski T.S., Alemany A., Parada G.E., Verity-Legg J. (2022). High-throughput total RNA sequencing in single cells using VASA-seq. Nat. Biotechnol..

[48] Pardo-Palacios F.J., Wang D., Reese F., Diekhans M., Carbonell-Sala S., Williams B. (2023). Systematic assessment of long-read RNA-seq methods for transcript identification and quantification. bioRxiv.

