# Supplementary material for: Single-cell profiling identifies LIN28A mRNA targets in the mouse pluripotent-to-2C-like transition and somatic cell reprogramming
Source: J Biol Chem. 2024 Sep 27;300(11):107824. doi: 10.1016/j.jbc.2024.107824 (PMC11584578; doi:10.1016/j.jbc.2024.107824)
Supplement: Supporting information [file mmc1.docx]

**Supporting information for:**

Single-cell profiling identifies LIN28A mRNA targets in the mouse pluripotent-to-2C-like transition and somatic cell reprogramming

Jieyi Hu^1,2,*^, Jianwen Yuan^3,4,5,*^, Quan Shi^6^, Xiangpeng Guo^7,8,9^, Longqi Liu^10,11^, Miguel A. Esteban^1,5,‡^, Yuan Lv^1,5,10,‡^

^1^Laboratory of Integrative Biology, Guangzhou Institutes of Biomedicine and Health, Chinese Academy of Sciences, Guangzhou, China

^2^University of Chinese Academy of Sciences, Beijing, China

^3^College of Life Sciences, University of Chinese Academy of Sciences, Beijing, China

^4^BGI Research, Shenzhen, China

^5^3DC STAR Lab, BGI CELL, Shenzhen, China

^6^Laboratory of Genomics and Molecular Biomedicine, Department of Biology, University of Copenhagen, Copenhagen, Denmark

^7^Centre for Genomic Regulation (CRG), Barcelona Institute of Science and Technology (BIST), Barcelona 08003, Spain

^8^Universitat Pompeu Fabra (UPF), Barcelona, Spain

^9^Bioland Laboratory (Guangzhou Regenerative Medicine and Health Guangdong Laboratory), Guangzhou, China

^10^BGI Research, Hangzhou, China

^11^Shanxi Medical University-BGI Collaborative Center for Future Medicine, Shanxi Medical University, Taiyuan, China

^*^These authors contributed equally to this work

^‡^For Correspondence: Miguel A. Esteban, [miguel@gibh.ac.cn](mailto:miguel@gibh.ac.cn); Yuan Lv, lvyuan@genomics.cn.

**Supporting figures:**

**Figure S1 (related to Figure 1).** Application of scTRIBE to mouse ESCs.

**Figure S2 (related to Figure 4).** Application of scTRIBE to somatic cell reprogramming.

**Supporting tables:**

**Table S1.** Statistics of significantly edited sites in mouse ESCs

**Table S2.** Differentially bound transcripts (DBTs) in different cell states of mouse ESCs

**Table S3.** Dynamic DBTs in somatic cell reprogramming

**Table S4.** Plasmids and primers used in this study

**Figure S1 (related to Figure 1). Application of scTRIBE to mouse ESCs.**

*A*, western blot showing expression of FLAG-tagged ADARcd and LIN28A-ADARcd induced by doxycycline in mouse ESCs. Ponceau S staining shows equal loading. DOX, doxycycline.

*B*, bar plot showing the number of all types of mutations in WT, ADARcd, and LIN28A-ADARcd mouse ESCs from bulk TRIBE results. Data are the mean ± s.d. of n = 2 biological replicates. *p* value was generated using a one-way ANOVA test. Except for A-to-G edits, no significant differences were detected across all other mutation types.

*C*, scatter plots showing correlation of gene expression between ADARcd and LIN28A-ADARcd from bulk TRIBE results. RPM, reads per million.

*D*, dot plot showing the editing frequency in WT, ADARcd, and LIN28A-ADARcd from bulk TRIBE results. Editing frequency was obtained by dividing edited reads by the total reads per edited site. Only significantly edited sites in LIN28A-ADARcd are plotted. *p* values were generated using a two-sided Wilcoxon test.

*E*, table showing details of the scRNA-seq datasets across ADARcd and LIN28A-ADARcd samples from mouse ESCs.

*F*, scatter plot showing correlation of normalized edited UMIs between replicates of LIN28A-ADARcd from scTRIBE results. The normalized edited UMIs were obtained by dividing edited UMIs by the total UMIs per transcript.

*G* and *H*, stacked bar plot showing the proportion of transcript types (*G*) and genomic region types (*H*) of total reads as identified by bulk TRIBE and scTRIBE.

**Figure S2 (related to Figure 4). Application of scTRIBE to somatic cell reprogramming.**

*A*, bar plot showing endogenous *Lin28a* mRNA expression level (relative to *Gapdh*) during reprogramming.

*B*, western blot showing the protein expression levels of LIN28A in sh*Luc*, sh*LIN28A* #1, and sh*LIN28A* #2 samples collected on D8 of reprogramming. ACTIN is the loading control.

*C*, bar plot showing cell number in sh*Luc*, sh*LIN28A* #1, and sh*LIN28A* #2 samples on D8 of reprogramming. Data are the mean ± s.d. of n = 2 biological replicates. *p* values were generated using a two-tailed Student’s *t*-test.

*D*, scanning micrograph showing the *Oct4*-GFP positive colonies in sh*Luc*, sh*LIN28A* #1, and sh*LIN28A* #2 samples on D8 of reprogramming. Data are the mean ± s.d. of n = 3 biological replicates. *p* values were generated using a two-tailed Student’s *t*-test.

*E*, western blot showing the protein expression levels of FLAG-tagged LIN28A-ADARcd induced by DOX on D6 and D8 of reprogramming. Histone 3 (H3) is the loading control.

*F*, table showing details of the scRNA-seq datasets from D6 and D8 reprogramming samples.

*G*, split UMAP plots from Fig. 4*B* showing the transcriptome of integrated cells of reprogramming spanning from D3 to D8 from Guo *et al.* and this study. Cells are colored by sample.

*H*, violin plot showing the number of edited UMIs (log_2_-transformed) in each sample. *p* value was generated using a two-sided Wilcoxon test.

*I*, violin plot showing the number of edited UMIs (log_2_-transformed) in each cell type (except ‘Other’). *p* value was generated using a two-sided Wilcoxon test.
