# Supplementary figures and images for: Single-cell profiling identifies LIN28A mRNA targets in the mouse pluripotent-to-2C-like transition and somatic cell reprogramming
Source: J Biol Chem. 2024 Sep 27;300(11):107824. doi: 10.1016/j.jbc.2024.107824 (PMC11584578; doi:10.1016/j.jbc.2024.107824)

Figure S1 (related to Figure 1)

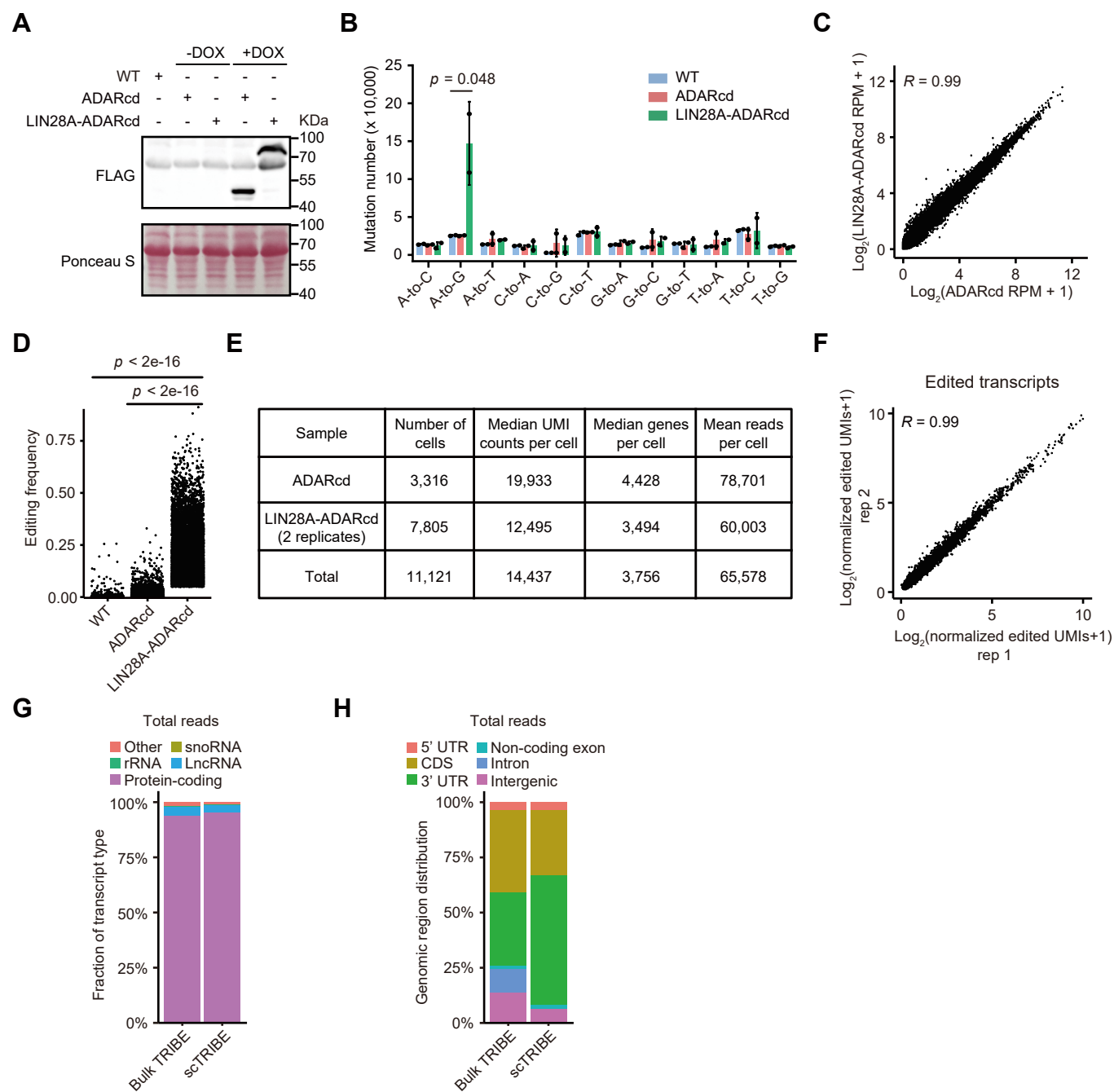

Supplement: Supplemental Figure S1 [file mmc6.pdf]

Figure S2 (related to Figure 4)

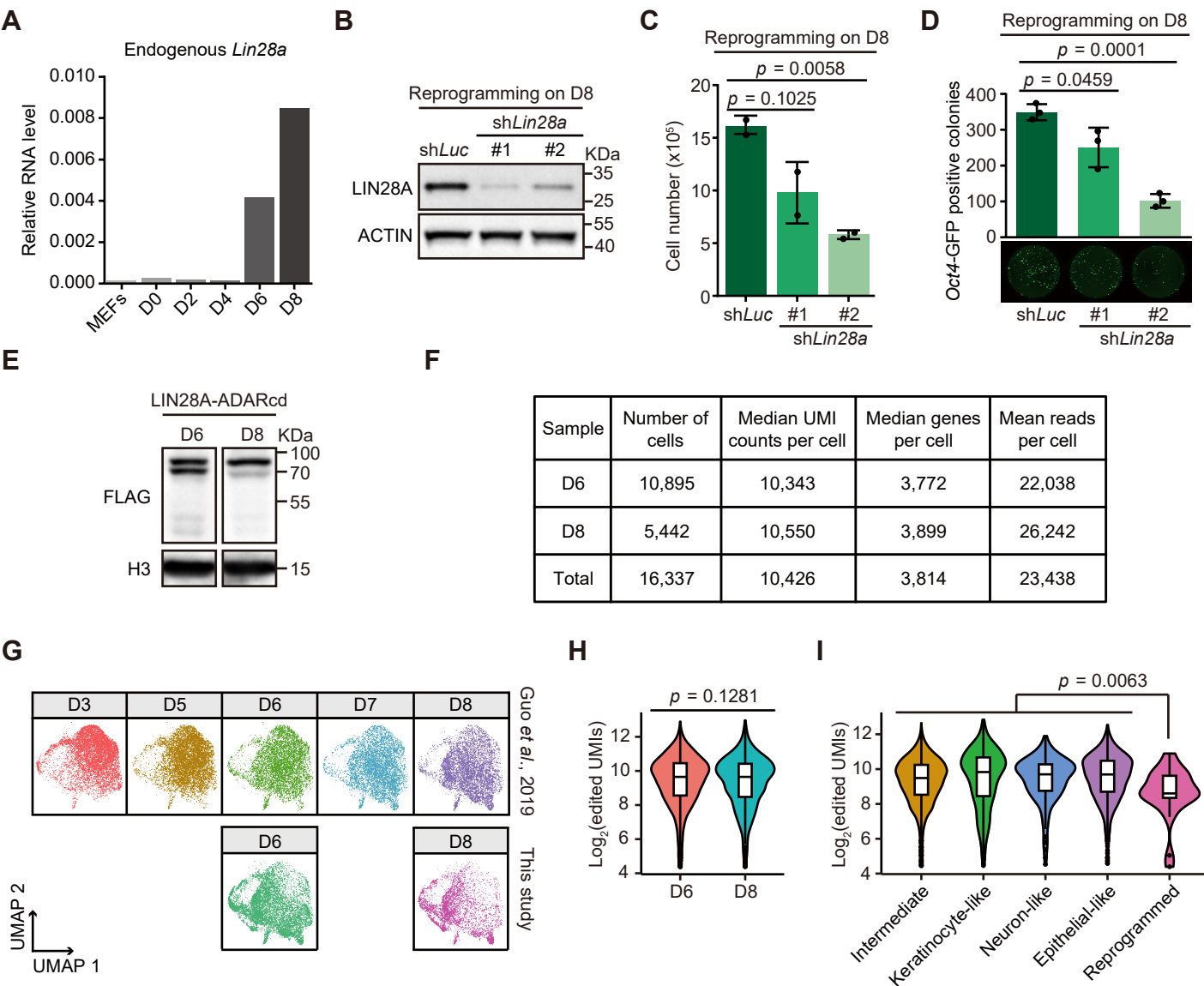

Supplement: Supplemental Figure S2 [file mmc7.pdf]
